# Supplementary material for: A Novel Bifidobacterium longum Subsp. longum T1 Strain from Cow’s Milk: Homeostatic and Antibacterial Activity against ESBL-Producing Escherichia coli
Source: Antibiotics (Basel). 2024 Sep 27;13(10):924. doi: 10.3390/antibiotics13100924 (PMC11505560; doi:10.3390/antibiotics13100924)
Supplement: Supplementary file 1 [file antibiotics-13-00924-s001.zip › antibiotics-3204570-supplementary.pdf]

Supplementary Materials

Table S1. Human primers used for qRT-PCR analysis of host immune response and homeostasis regulation by BLLT1.

Primer	Sense Primer	Antisense Primer	Reference
TLR4	TGCACAGGACAGAACATCTCTGGA	AGCTCCTGCAGGGTATTCAAGTGT	[169]
IAP	CATACCTGGCTCTGTCCAAGA	CGCTCCACCAACTAAGAACG	[170]
β-actin	TCACCCACACTGTGCCCATCTACGA	CAGCGGAACCGCTCATTGCCAATG	[169]
